# Do 6-8 year old girls with central precocious puberty need routine brain imaging?

**DOI:** 10.1186/s13633-016-0027-5

**Published:** 2016-05-04

**Authors:** Paul B. Kaplowitz

**Affiliations:** Division of Endocrinology, Children’s National Health System, Washington, DC USA

**Keywords:** Precocious puberty, Brain tumor, Astrocytoma, Glioma, Brain imaging, Pituitary microadenoma

## Abstract

**Background:**

The subject of whether all girls with central precocious puberty (CPP) require brain imaging is controversial.

**Findings:**

A review of the major papers concerning this topic published since 1994 was conducted looking primarily at the frequency of occult intracranial lesions, particularly brain tumors, in girls with CPP. While CNS abnormalities are frequently noted (8–15 %), the proportion of previously unknown findings requiring intervention in 6–8 year old girls is very small, in the range of 0–2 %.

**Conclusion:**

While MRI should still be done in boys and in girls with onset of puberty younger than age 6 and in boys, ordering an MRI should not be routine in 6–8 year old girls with CPP. Suggestions are made as to how to approach the decision-making process with the parents regarding brain imaging in asymptomatic 6–8 year old girls with CPP.

Although there is much evidence that female puberty is starting earlier now than in the past [1, 2], the widely accepted definition of central precocious puberty (CPP) remains onset in girls before age 8 and in boys before age 9. It is considered essential by many pediatric endocrinologists to order brain imaging in all children with CPP to rule out the possibility of a CNS lesion such as a brain tumor which might require intervention. This commentary will explore some of the findings which have been used to support this and why it might be time to revise this recommendation, at least for girls presenting with CPP at greater than 6 years of age.

It has long been known that most cases of girls with CPP are idiopathic, and estimates during the 1970s and 1980s put this figure at about 80 %. With the widespread use of CNS imaging beginning in the early 1980s, small occult CNS lesions could be detected in asymptomatic girls with CPP, leading to concerns that these lesions, including tumors such as astrocytomas and gliomas, might be missed unless all girls with CPP underwent brain imaging. However, in a 1995 report, Bridges et al at Middlesex Hospital in London [3] reported finding no small asymptomatic lesions in their series of 91 girls with CPP, 6 of whom had previously diagnosed intracranial pathology (2 with tumors), and concluded that obtaining brain imaging on all girls with CPP could not be justified.

A larger series of patients in a 2000 European study involving 304 girls with CPP who underwent brain imaging found that 18.4 % had a CNS lesion, of which half were previously known. Of the CNS lesions found, 8.5 % were newly discovered, though the number involving neoplasms was smaller [4]. Interestingly, the authors reported no significant age-related differences in the chances of finding a CNS abnormality, with 7 % of girls between ages of 7 and 8 having a CNS finding. A 2002 study from France [5] asked the question of whether the 1999 article based on the 1997 PROS study, which proposed a cut-off of 7 years for when puberty should be considered precocious and require evaluation [6], would result in missing important CNS lesions. The French study looked at 197 girls with CPP evaluated from 1982 to 2000 and found 11 (6 %) with a CNS abnormality, but age of onset was a critical factor; in girls with onset under age 6, 19 % had a CNS lesion whereas between the ages of 6 and 8, the incidence was only 2 %. The authors sought to develop a predictive model which could identify a group of girls at such low risk that CNS imaging could be safely avoided. Using this model, they reported that age <6 years or estradiol >45^th^ %ile identified all girls with occult intracranial lesions (OCIL). When the model was applied to a larger group of 443 girls from seven centers in six European countries [7], the results were similar: 98 % of girls with pubertal onset after age 6 had idiopathic CPP while 2 % had an OICL, but for the group with puberty onset before age 6, 29 % had an OICL. Using an estradiol cutoff of >45^th^ %ile for girls with CPP according to the distribution of values at each center identified all 6 girls with onset over age 6 who had an OCIL, but of those who had an estradiol >45^th^ %ile, only 3 % had an OCIL, still a small proportion. In an editorial accompanying this article, Stanhope reviewed the existing literature on occult intracranial tumors in CPP and concluded that “the previous recommendation of denying neuroimaging to girls with CPP made by Bridges et al can no longer be substantiated. It is unfortunate to miss the diagnosis of an early and treatable hypothalamic tumor, such as an astrocytoma, especially as magnetic resonance imaging does not involve ionizing radiation” [8].

One problem with the approach of using estradiol as a key part of decision-making for ordering brain imaging is that it would still require that imaging be done on nearly half of girls with CPP. Furthermore, estradiol levels are notoriously difficult to interpret. Unlike in males where consistently rising testosterone levels during puberty allow one to estimate how far along in the pubertal continuum a boy falls, estradiol levels are less reliable. Even with the more sensitive assays which can detect levels as low as 5 pg/mL, it is not unusual to find girls who are clearly progressing though puberty with pubertal LH values but estradiol levels which are not elevated, as well as estradiol levels in the range of 30 pg/mL in girls who during follow-up prove to have premature thelarche, not CPP. Also given that different labs use different assays for estradiol, it would fall upon every center to pool enough results to determine their 45^th^ %ile cut-off to discriminate idiopathic CPP from CPP due to an OICL, a difficult task.

In 2007, a conference including pediatric endocrinologists from the North American and European societies was held to produce a consensus paper on the use of GnRH analogues in children. One of the topics addressed was the need for brain imaging in CPP, which appears to be routine in most European centers but is not as consistently recommended for 6–8 year old girls in US centers. The consensus paper [9] acknowledges this, and stated.

CPP may be a sign of central nervous system pathology. Unsuspected intracranial pathology has been reported in 8 % of girls and 40 % of boys without neurologic findings or neurofibromatosis. The percentage of children with unsuspected intracranial pathology decreases with age. Only 2 to 7 % of girls who have onset of CPP between the ages of 6 and 8 years have unsuspected pathology, and only ∼ 1 % have a tumor such as a glioma or astrocytoma. Factors that may decrease the likelihood of finding a tumor include racial/ethnic background, family history of CPP, and adoption.

Conclusions: All boys with CPP and girls with CPP at <6 years of age should have a head MRI. *It is controversial whether all girls who develop CPP between 6 and 8 years of age require head MRI.* Girls with neurologic findings and rapid pubertal progression are more likely to have intracranial pathology and require an MRI examination (BII).

A 2012 study from Copenhagen reviewed imaging findings in 229 girls with CPP evaluated during a 16 year period between 1993 and 2009 who underwent a brain MRI [10]. Of these, 54 had an abnormal study but 21 were previously known findings, 20 were categorized as incidental findings including pineal cysts and four pituitary microadenomas, and 13 had new findings on MRI, which included five arachnoid cysts, two hypothalamic hamartomas, and only one (0.5 %) newly discovered tumor requiring surgery, a pilocytic astrocytoma. Nonetheless, the authors of this study concluded that “A high frequency of 6–8 year old girls with precocious puberty in our study had a pathological brain MRI, which could not be predicted from any clinical nor biochemical parameters. Thus, we believe that girls with precocious pubertal development of central origin before 8 years of age should continue to be examined by a brain MRI”.

The most recent study on this topic published in December 2014 presents findings in 182 girls with breast development onset before age 8 and a diagnosis of CPP evaluated at the Bambino Gesu General Hospital in Rome between January 1990 and December 2012 [11]. All had MRIs with a detailed examination of the hypothalamic-pituitary area. Normal MRIs were found in 157 (86 %) while in 14 (8 %) incidental abnormalities were found, 5 had microadenomas, and only 6 (3 %) had pathological abnormalities, all hamartomas with no tumors requiring surgery. What is important is that all the girls with hamartomas had onset of puberty before age 6 (mean age 1.87 years), and had higher baseline and stimulated LH, FSH and estradiol than girls with idiopathic CPP. There was no difference in LH levels between the five girls with microadenomas and those without; also the microadenomas that were reimaged did not increase in size and one disappeared. The authors also pointed out the importance of a family history of CPP, as 46 of the girls in their study had a mother or other relative with menarche at age 10 or younger, and none had a hamartoma or other important CNS finding. The authors therefore questioned whether routine MRI is needed in girls older than age 6 with CPP, particularly those with a family history, and concluded that better evidence-based criteria to help with clinical decision making were needed.

So how should we use the ample data from the studies cited above to inform the decision as to whether to order brain imaging in patients with CPP? There is no argument that all boys with CPP, girls with CPP with onset before age 6, and any girl with clinical findings indicating the possibility of a CNS lesion (e.g. severe frequent headaches, changes in vision or new onset seizures) need brain imaging as the yield of new findings which may affect clinical management is significant. The real issue is what to do about girls with CPP onset between ages 6 and 8 with no CNS-related symptoms, who form the great majority of CPP patients seen by pediatric endocrinologists. While rare girls in this category will harbor an OICL, we should also consider the costs of doing so many brain MRIs or CTs to find such a small number (estimated to be in the range of 1 %) of tumors such as astrocytomas and gliomas which will require surgery. (It should be acknowledged that there is no consensus as to how frequent a serious finding must be to justify expensive imaging studies for many conditions in medicine). Although brain MRIs do not expose the child to ionizing radiation, they are significantly more expensive than CT scans. The average charge in the US in 2014 was $2600, which does not include the charge for the neuroradiologist interpreting the study, as well as sedation which is often needed in children, and IV administration of contrast agents, both of which increase the risk of the procedure. There is also the consideration that ordering a brain MRI increases parental anxiety and may be quite stressful for the child. Furthermore, doing MRIs on girls with CPP at low risk for OICL will result in picking up minor incidental findings like pituitary microadenomas, which as pointed out [11], are very unlikely to be related to CPP. They rarely, if ever, increase in size during follow-up exams but may result in additional lab testing, a visit to a neurosurgeon and in many cases at least one more MRI.

Part of the reason the incidence of tumors in 6–8 year old girls with CPP is so low in recent studies may be that the incidence of CPP in this age group has increased, as part of a larger trend towards earlier puberty in girls, the cause of which has been much debated but is likely due at least in part to the obesity epidemic [12]. The study from Copenhagen of 449 girls evaluated for early puberty between 1993 and 2007 found a significant increase after 2003 of both CPP onset before age 8 and early puberty onset between the ages of 8 and 9 [13]. As we are seeing more healthy (but often heavy) girls maturing earlier, it is perhaps not surprising that the proportion with OICL, which was never large, has become very small.

One issue many of us grapple with is the legal implications of missing a brain tumor in a child with CPP. My approach to this is to point out to the parents that the risk of finding a tumor in an asymptomatic 6–8 year old girl with CPP is at most 1 %, and that I would not recommend brain imaging given that small risk. I have found that most parents do not push for imaging when it is presented that way, but if they cannot live with even that small amount of uncertainty, then it makes sense to order the MRI. However, if they are informed of the small risk and included in the decision-making process, I believe that the chance of medicolegal challenges in the unlikely event that a CNS tumor is eventually found is reduced significantly. We are all well-intentioned providers wanting to provide the best care for our children and their families, but in this situation, routine MRIs in all girls with CPP may not be the optimal approach. Until we have more reliable guidelines to identify the rare child with CPP and an OICL, it may be best to review the available evidence with the parents and get their input before making a final decision on brain imaging.

## Conclusion

Considering the very low frequency of finding brain tumors which require intervention in 6–8 year old girls with CPP and no CNS-related symptoms, it is proposed that brain imaging not be ordered routinely but discussed with the family after explaining both the benefits and risks as noted above.
